# Profil de l'hémogramme chez les enfants paludéens de 0 à 5 ans sous quinine, cas de la République Démocratique du Congo

**DOI:** 10.11604/pamj.2014.19.95.5174

**Published:** 2014-09-26

**Authors:** Arsène Tshikongo Kabamba, Zet Kalala Lukumwena, Albert Otshudi Longanga

**Affiliations:** 1 Faculté des Sciences Pharmaceutiques, Université de Lubumbashi, République Démocratique du Congo; 2 Faculté de Médecine Vétérinaire, Université de Lubumbashi, République Démocratique du Congo; 3 Université Libre de Bruxelles (ULB), Bruxelles, Belgique

**Keywords:** Hémogramme, paludisme grave, enfants, quinine, Hemogram, severe malaria, infants, quinine

## Abstract

Le paludisme constitue un des problèmes de santé publique majeur en République Démocratique du Congo (RDC) à cause d'une part des risques d’épidémies dans certaines zones du pays et d'autre part à cause du nombre des malades et des décès qu'il provoque. Cette étude expose les aspects hématologiques liés à la prise de la quinine au cours du paludisme grave chez l'enfant. Pour ce faire, les prises de sang ont été effectuées à deux groupes d'enfants, dans différents centres hospitaliers de Lubumbashi: le premier groupe est constitué d'enfants gravement impaludés et sous traitement à la quinine; tandis que le second groupe, composé d'enfants impaludés aussi mais sans traitement à la quinine et sert de groupe témoin. Ces prélèvements ont été analysés pour une exploration de l'hémogramme par un dosage sérique des paramètres hématologiques ci-après: les globules rouges, l'hémoglobine, l'hématocrite et le volume globulaire moyen. Les résultats obtenus montrent une différence statistiquement significative entre les deux groupes d'enfants examinés. En effet, dans la majorité des cas, une augmentation des taux plasmatiques des paramètres hématologiques analysés a été observée dans le groupe d'enfants impaludés sous traitement à la quinine, traduisant ainsi l'apport de la quinine sur la stabilisation de l'hémogramme au cours d'un paludisme grave chez l'enfant de moins de cinq ans.

## Introduction

Le paludisme est une érythropathie parasitaire due à des protozoaires du genre plasmodium. C'est de loin la parasitose qui touche le plus grand nombre de personnes et provoque le plus de décès [1]. Il traduit l'agression directe du globule rouge et sa destruction par le plasmodium; il est à la base de l'hémolyse dite intra-vasculaire et d'autre part, la destruction par érythrocytose des globules rouges infectés ou non par le système réticulo-endothélial entrainant l'hémolyse dite extra-vasculaire [2, 3].

Selon les dernières estimations de décembre 2013, l'OMS a enregistré, en 2012, 207 millions de cas de paludisme qui ont causé 627 000 décès. La plupart de décès surviennent chez des enfants vivant en Afrique où chaque minute un enfant meurt du paludisme. En Afrique le taux de mortalité des enfants a diminué de 54% par rapport à l'an 2000 [4]. Malgré cette régression visible de cette parasitose au cours des dernières années dans toutes les régions du monde, les chiffres de morbidité et de mortalité restent toujours alarmants et fait du paludisme la maladie parasitaire tropicale la plus importante. Elle concerne 36% de la population mondiale dans plus de 90 pays ou territoires. Plus de 90% des cas et des décès concernent l'Afrique sub-saharienne, en particulier chez les enfants de moins de 5 ans [5, 6].

Les enfants de moins de 5 ans représentent l'un des groupes les plus touchés par cette parasitose sous sa forme grave avec des complications hématologiques innombrables et dont l'anémie qui est la composante la plus importante du paludisme grave; cause de la morbidité chez les enfants dans les pays d'endémie palustre [7, 8]. Dans les zones à forte transmission, une immunité partielle à la maladie est acquise pendant l'enfance. Dans de tels environnements, la majorité des cas de paludisme, en particulier les cas sévères progressant rapidement vers le décès, sont observés chez de jeunes enfants qui n'ont pas acquis d'immunité. Une anémie sévère, une hypoglycémie et un paludisme cérébral sont des caractéristiques du paludisme severe plus fréquemment observés chez les enfants que chez les adultes [4, 9]. Ce travail se propose d’étudier l'anémie due à l'infestation palustre; vise également à évaluer les perturbations de l'hémogramme observées au cours de la crise du paludisme en cours de traitement à la quinine; il voudrait aussi apprécier l'importance des perturbations hématologiques en relation avec la prise de la quinine.

## Méthodes

L’échantillon qui a fait l'objet de notre étude est constitué des petits enfants souffrant du paludisme grave sous traitement à la quinine et des enfants souffrant du paludisme sans traitement à la quinine. Les critères de sélection d'enfants malades et témoins sont ceux fixés par l'OMS pour le paludisme grave à savoir: troubles de conscience, convulsions répétées, prostration (extrême faiblesse), détresse respiratoire, ictère, hémoglobinurie macroscopique, collapsus circulatoire, œdème pulmonaire, saignement anormal, anémie grave, hypoglycémie, acidose métabolique, hyperparasitémie. La présence chez l'enfant impaludé d'un seul critère parmi ceux énumérés ci-dessus est suffisante pour le diagnostic du paludisme grave [4].

Le premier groupe (groupe I) est constitué de 23 enfants de moins de 5 ans, hospitalisés dans les différents centres hospitaliers de Lubumbashi: Cliniques universitaires, polyclinique Medicare et polyclinique saint-Luc. Le deuxième groupe (groupe témoin) est constitué de 23 enfants impaludés aussi répartis dans les communes de Kampemba, Kamalondo et Kenya de la ville de Lubumbashi. Leur participation à l’étude était conditionnée par un accord préalable de leurs parents ou de leurs responsables.

Nous avons évalué l'hémogramme par la mesure sérique des globules rouges, Hémoglobine, Hématocrite et volume globulaire moyen dans les échantillons prélevés dans les deux groupes, selon les valeurs normales [10, 11]. Les résultats obtenus ont été saisis et analysés sur le logiciel Epi Info 2011 (version 7.0.8.1). Les moyennes sont présentées avec les écarts-types, avec un intervalle de confiance à 95% (IC 95%). Le test de Student a été utilisé pour la comparaison des moyennes et celle des fréquences (exprimées en pourcentage) par le test de X2 corrigé de Yates ou le test exact de Fisher lorsque recommandés. Le seuil de signification a été fixé à p

## Résultats

Les résultats des différents paramètres analysés sont repris dans le Tableau 1. La taille de l’échantillon étant égal à 23 (n=23), la valeur obtenue pour chaque paramètre dosé correspond à la moyenne plus l’écart-type. Le test t de Student a été utilisé pour permettre de comparer les résultats obtenus dans les deux groupes d'enfants (P = 0,000).

**Tableau 1:** Évaluation de l'hémogramme: enfants impaludés sous quinine versus enfants impaludés sans quinine

Paramètres dosés	Groupe I (n =23)	Groupe II (n = 23)	Valeurs de référence
Hémoglobine (g/dl)	12,46 ± 1,32	10,3 ± 1,41	11,1-15,13 g/dl
Globules Rouges	3953913,04 ± 545 966,52	3724347,82 ± 464 687,83	4,00-5,40 millions/mm^3^
Hématocrite	35,73 ± 3,25	31,56 ± 3,16	36-45%
VGM	88,39 ± 10,22	80,05 ± 7,71	74 – 92 fl

## Discussion

Une différence statistiquement significative a été observée à l'issue du dosage de quelques paramètres indispensables à l’évaluation de l'hémogramme entre le groupe d'enfants impaludés sous traitement à la quinine et celui d'enfants impaludés sans traitement à la quinine.

Premièrement, environ 89% d'enfants de groupe I ont un taux d'hémoglobine élevé contre 31% d'enfants du groupe II (t=11,2150; p=0,0000). Ceci pourrait être dû à un état d'hyper-hémolyse qui survient pendant la phase de schizogonie érythrocytaire chez l'enfant impaludé sans traitement à la quinine; par contre l'augmentation chez l'enfant impaludé sous traitement à la quinine révèle l'apport de la quinine dans la stabilisation des hémolyses. Ceci corrobore les résultats de Nambei et al. (2013) qui ont prouvés l'efficacité de la combinaison des antipaludéens dans le traitement de diverses complications dont les complications hématologiques [8]. Le taux d'hémoglobine est un des paramètres indispensables pour affirmer avec précision la présence ou non d'une anémie. Une des formes graves du paludisme est la forme anémique. Il faut noter que nos enfants congolais qui ont au départ une anémie relative à une malnutrition et aux multiples parasitoses, présentent des anémies profondes lors d'une hyper-parasitemie paludique. Le taux d'hémoglobine tombe alors brutalement en dessous des valeurs acceptables. Un collapsus peut survenir; la solution la plus urgente est de transfuser l'enfant avec du culot globulaire tout en soutenant le cœur avec des tonicardiaques [1, 2].

Deuxièmement, nous avons constaté également une élévation significative des globules rouges dans le groupe d'enfants malades sous traitement à la quinine (84%) par rapport aux enfants sains (31%). Ces données corroborent les observations faites au cours des études réalisées antérieurement par Gansane et al. (2013), Bougouma et al. (2012) qui indiquent que dans tous les cas de paludisme graves, l'anémie est une conséquence inévitable par les hémolyses excessives. D'autre part, une élévation modérée du taux de globules rouges peut refléter une discrète inhibition des anémies hémolytiques dues au plasmodium et une production d'autres cellules par la moelle osseuse [1–3]. Nous pensons que l'accroissement significatif des taux des globules rouges dépend de la parasitémie et du degré de production des cellules sanguines par la moelle osseuse et que cette relation est fonction du temps après infestation. Ainsi, l’élévation du taux des globules rouges est un indicateur non seulement d'une inhibition de l'hémolyse mais aussi de la normalisation de l'hémogramme par la quinine [7].

Toutefois, la numération érythrocytaire de l'enfant africain vivant en milieu tropical et exposé à diverses parasitoses et à la malnutrition est d'un niveau habituellement bas. Ce qui laisse alors penser à une origine mixte de l'anémie. Par ailleurs l'anémie sévère peut se constituer à la suite d'un accès palustre grave avec parasitémie élevée, mais peut aussi résulter d'une hémolyse chronique par suite d'accès palustres. Ce qui pourrait donner lieu dans le second cas à une anémie intense même avec une parasitémie faible.

Troisièmement, nos analyses ont également révélé une élévation significative des taux de l'hématocrite chez les enfants paludéens sous traitement à la quinine. Nous pensons que la mesure de ce paramètre hématologique est justifiée dans le cas d'un paludisme grave car comme l'indiquent Gansane et al. (2013), dans le cadre d'une évaluation des paramètres hématologiques chez les enfants paludéens de moins de 5 ans, la mesure de l'hématocrite constitue un meilleur indicateur de l’état de l'hémogramme après hémolyses. La baisse de l'hématocrite n'est pas inattendue puisqu'elle définit même l'anémie.

Quatrièmement, nous avons constatés, une augmentation du volume globulaire moyenne chez les enfants paludéens sous traitement à la quinine. Le volume globulaire moyen renseigne sur le type d'anémie; nous réalisons que le taux était normal malgré l'augmentation. Nous pensons donc que lors du paludisme, on assiste à une anémie normochrome. Ceci corrobore les résultats de Gansane et al. (2013) et Bougouma et al. (2012) qui montrent que les anémies normochromes sont habituellement rencontrées au cours des hémorragies aigues, des anémies hémolytiques comme dans le cas du paludisme, et de certaines aplasies [1, 9].

## Conclusion

Dans la présente etude, nous avons évalué l'hémogramme au cours du paludisme grave chez les enfants de moins de 5 ans sous quinine et nous concluons à la lumière des résultats de notre étude que la quinine est capable de stabiliser les perturbations hématologiques causées par le paludisme grave à Plasmodium falciparum. Ces perturbations résultent vraisemblablement des hémolyses excessives enregistrées lors du paludisme grave chez les enfants de 0 à 5 ans.
